# Extraction and characterization of natural fibers from *Pulicaria gnaphalodes* plant and effect of alkali treatment on their physicochemical and antioxidant properties

**DOI:** 10.3389/fchem.2024.1437277

**Published:** 2024-08-02

**Authors:** Mohammed Alsafran, Kishor Kumar Sadasivuni, Julna Mohammed Haneesh, Deepak M. Kasote

**Affiliations:** ^1^ Agricultural Research Station, Qatar University, Doha, Qatar; ^2^ Center for Advanced Materials, Qatar University, Doha, Qatar; ^3^ Department of Mechanical and Industrial Engineering, Qatar University, Doha, Qatar

**Keywords:** *Pulicaria gnaphalodes*, natural fiber, alkali treatment, antioxidant activity, lignocellulosic

## Abstract

The study aimed to extract and characterize natural fibers from *Pulicaria gnaphalodes* (Vent.) Boiss. plants and assess the impact of alkali treatment on the physicochemical and antioxidant properties of these fibers. Fibers were extracted from dried *P. gnaphalodes* aerial parts by grinding with an average yield of 18.1%. Physicochemical and FTIR analysis revealed that the hemicellulose was mostly lost during alkali treatment. Results of the X-ray diffraction and thermogravimetric analysis indicated that the crystallinity and thermal stability of *P. gnaphalodes* fibers were considerably increased after alkali treatment. In antioxidant activity assessment studies, raw fibers of *P. gnaphalodes* showed significantly higher radical scavenging and reducing power potentials compared to the alkali-treated samples, indicating that the majority of antioxidant components such as lignin and other polyphenols were lost from *P. gnaphalodes* fibers during alkali treatment. In conclusion, the promising antioxidant activity of raw *P. gnaphalodes* can be utilized in developing functional materials, particularly for cosmetic and wound healing applications.

## 1 Introduction

Non-woody plant fibers are generally termed natural fibers, which are rich in cellulose and have good physicochemical properties and crystallinity (Gholampour and Ozbakkaloglu, 2020). Moreover, their affordability, environmental friendliness, and widespread availabilities make them popular materials in diverse industries including household goods, marine applications, and automotive sectors (Lokantara et al., 2020). Likewise, raw plant fibers such as sisal and ramie fibers are also found to be useful for biomedical purposes due to their biocompatibility and biological properties (Kandimalla et al., 2016; Guambo et al., 2020; Zamora-Mendoza et al., 2022). However, the use of plant fibers in their raw form has various limitations, as they possess high moisture absorption, poor durability, as well as low thermal stability and strength (Zamora-Mendoza et al., 2023). Hence, modifying surfaces of natural fibers is usually recommended to increase their crystallinity, hydrophobicity, mechanical, and thermal properties (Tavares et al., 2020).

Various physical, chemical, and biological methods have been employed to modify natural fiber surfaces (Cruz and Fangueiro, 2016). Physical treatments such as plasma, γ-ray treatments, and corona treatment reacts with surface functional groups of the fibers and modify them, thereby significantly improving the mechanical properties of natural fibers (Jagadeesh et al., 2021). Similarly, natural fibers have also been treated with various chemicals such as alkali, silane, water-repelling agents, peroxides, and permanganates to improve the crystallinity, hydrophobicity, mechanical, and thermal properties of natural fibers (Cruz and Fangueiro, 2016). Moreover, besides physical and chemical treatments, biological methods mainly using enzymes are also used to treat natural fibers (Koohestani et al., 2019). However, there is a need to understand the effect of these treatments on the surface, mechanical, and biological properties of different natural fibers.

*Pulicaria gnaphalodes* (Vent.) Boiss. is one of the commonly occurring *Pulicaria* species in Qatar, locally known as “Nufaij” (Abdel-Bari, 2012; Kasote et al., 2024). It is a perennial or subshrub that primarily grows in the temperate biome and is native to Iraq, Central Asia, the Western Himalayas, and the Arabian Peninsula (Kasote et al., 2024; POWO, 2024). However, *P. gnaphalodes* is reported to be a widely distributed species in the Persian region and is recognized as a medicinal plant (Hassanabadi et al., 2023). Traditionally, this plant is used as an herbal tea, including as a flavoring, antimicrobial, and anti-inflammatory agent (Kazemi et al., 2022). In recent studies, most of these traditional uses have been validated, and *P. gnaphalodes* has been reported to have promising antioxidant, antimicrobial, anticancer, antihypercholesterolemic, and anticonvulsant properties (Kamkar et al., 2013; Naqvi et al., 2020; Zadali et al., 2022; Pourhossein Alamdary et al., 2023). Flavonoids, terpenes (monoterpenes, sesquiterpenes, diterpenes, and triterpenes), and phenolic acids are the main reported bioactive phytochemicals in *P. gnaphalodes* (Kasote et al., 2024). As previously reported, *Pulicaria undulata* (L.) CA Mey. is known to be rich in fiber (Fahmi et al., 2019). We also found that the aerial part of *P. gnaphalodes* is rich in fibers. However, fibers from *P. gnaphalodes* have not yet been extracted and characterized, and their potential bioactivity also remains unexplored.

Herein, we extracted fibers from *P. gnaphalodes* for the first time and studied their physicochemical, thermal, and surface properties. Similarly, the effect of alkali treatment on the matrix and surface of *P. gnaphalodes* fibers was also investigated. Moreover, the antioxidant potential of both raw and alkali-treated fiber samples of *P. gnaphalodes* was evaluated to understand their potential wound healing and cosmetic applications.

## 2 Material and methods

### 2.1 Materials

Plants were harvested from Ash Shamal, Qatar. The aerial parts were cleaned, separated from the roots, and dried at 50°C for 72 h in a hot air oven to extract fibers. ABTS [2,2′-azinobis (3-ethylbenzothiazoline-6-sulfonic acid)] was purchased from Sigma-Aldrich, China. All other chemicals used, including potassium permanganate, potassium persulfate, sodium hydroxide, acetone, ethanol (95% pure), sulfuric acid, and ammonium oxalate, were of analytical grade.

### 2.2 Extraction of fibers

**FIGURE 1:**
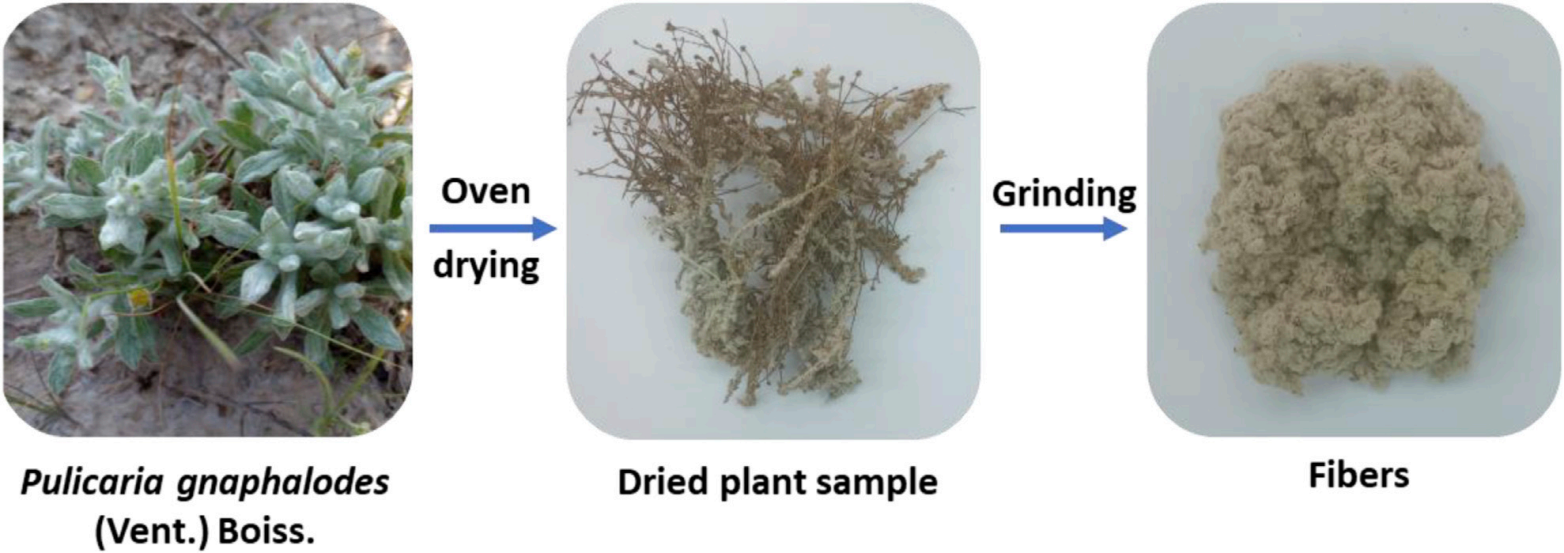
The workflow of fiber extraction process from *P. gnaphalodes*.

Fibers from *P. gnaphalodes* were extracted by grinding the dried aerial parts in a home-use grinder. The workflow of fiber extraction from *P. gnaphalodes* is summarized in Figure 1. The fibers were manually cleaned before conducting physicochemical analysis and characterization studies. The fiber yield was then measured.

### 2.3 Alkali treatment

An aliquot of 20 mL of 0.2N NaOH (5%) was added to the Falcon tube containing around 1 g of raw fiber sample and the tube was kept in a boiling water bath for 90 min. The tube was cooled for 1 h before neutralizing the reaction mixture with a 1% (w/v) HCl solution. The fiber sample was then washed with distilled water after centrifugation at 5,000 rpm for 5 min. This washing process was repeated two to three times until the fiber sample was neutralized. The neutralized fiber sample was dried in an air oven at 105°C for 24 h and stored in Ziploc bags until further analysis (Ilangovan et al., 2018; Senthamaraikannan and Saravanakumar, 2023).

### 2.4 Physicochemical analysis

#### 2.4.1 Physical analysis

In the physical analysis, the diameters of the raw and alkali-treated fibers were measured using scanning electron microscopy (SEM). The diameter of each 3–6 single fiber was measured at 3–4 locations on each fiber, and the average value was recorded for each fiber.

#### 2.4.2 Chemical analysis

Cellulose, hemicelluloses, lignin, extractives, wax, ash, and moisture content in raw and alkali-treated fibers were determined using different methods. The moisture content was initially determined using a method described by Ngoup et al. (2024). Ash content in fiber samples was determined using the ASTM E1755-01 Method (Kulandaivel et al., 2018). To estimate the wax content, 1 g ± 0.1 g of both raw and treated fiber samples (n = 3) were placed in 50 mL of hot ethanol for 3 h. After extraction, the samples were dried overnight at 105°C and reweighed. The wax content was estimated based on weight loss (Thomsen et al., 2005). The lignin content of raw and treated fibers was measured using the gravimetric method (Valls Calm, 2017). Dewaxed fiber samples (100 ± 0.1 mg, n = 3) were placed in Falcon tubes and treated with 3.0 mL of 72% sulfuric acid for 24 h. The samples were then diluted with 18 mL of water and hydrolyzed for an additional 5 h. The lignin content was determined gravimetrically after the hydrolysis process (Valls Calm, 2017). To determine the extractives content, 20 mL was added to dewaxed samples (200 ± 0.1 mg) and kept in a water bath at 56°C for 1 h. After centrifugation, the acetone extract was separated, and the acetone was allowed to evaporate. The weight of the remaining residue was then measured to calculate the percentage of extractives content based on the weight loss (Kale et al., 2018). The pectin content was estimated using the dried residues of fiber samples after extractives removal. An aliquot of 20 mL aliquot of a 0.5% solution of ammonium oxalate was added to the fiber residues and heated in a boiling water bath for 5 h. The supernatant was then separated, dried, and weighed after cooling, and the percentage of pectin was calculated based on the weight loss (Whistler et al., 1940). The remaining residue was used for the estimation of hemicellulose and cellulose after weighing. To estimate hemicellulose, 10 mL of 0.5N NaOH was added to the tube containing the fiber sample residues left after pectin isolation, and these tubes were placed in a boiling water bath for 3 h. Following centrifugation (5,000 rpm, 5 min), the residue was collected, washed with water, and then dried in the oven. The weight of this residue was measured, and the weight percentage was calculated as the hemicellulose content (Cakmak and Dekker, 2022). Finally, the cellulose content (%) was calculated using the formula “100-%wax-%extractives-%pectin-%hemicellulose-%lignin” (Alsafran et al., 2024).

### 2.5 Fourier transform-infrared (FTIR) analysis

FTIR analysis was conducted to identify the functional groups in the fiber samples. The raw and alkali-treated fibers were powdered, mixed with KBr, and pressed into pellets. Spectra were obtained using a Perkin Elmer Spectrometer in the range of 4,000–500 cm^−1^.

### 2.6 X-ray diffraction (XRD) analysis

The PANalytical Empyrean X-ray diffractometer (Malvern Panalytical B.V., Brighton, United Kingdom; Cu Kα = 1.5404 Å) was used to estimate the crystallinity index of raw and alkali-treated fiber samples. Scans were measured at 2θ in the range of 10°–80° with an accuracy of 0.02.

### 2.7 Thermogravimetric analysis (TGA)

Thermal stability of raw and alkali-treated fiber samples was measured using thermogravimetric analysis (PerkinElmer, Pyris 6, United States). Fiber samples were placed in an alumina crucible and then kept in the furnace with a controlled environment of nitrogen flow rate of 20 mL/min. The temperature of the chamber was increased from room temperature to 600°C at a rate of 10°C/min.

### 2.8 Scanning electron microscopy (SEM) analysis

The surface topography and diameters of raw and alkali-treated fibers were analyzed using SEM. The SEM images and energy dispersive X-ray spectroscopy (EDX) data were obtained with a JCM 6000 SEM.

### 2.9 Antioxidant activity

The antioxidant and reducing power properties of raw and alkali-treated fiber samples were evaluated using modified ABTS and potassium permanganate (KMnO_4_) reduction (PPR) assays described by Kasote et al. (2019). In the ABTS assay, 7.4 mM ABTS and 2.6 mM potassium persulfate solutions were prepared and mixed in equal amounts (v/v) and left to react overnight in the dark. The next day, the reagent was diluted with water (1:40, v/v) before use. Fiber samples (10 ± 0.1 mg, n = 4) were placed in 24-well plates, and 1 mL of the reagent solution was added to each well, including control wells. The plate was incubated for 5 min in the dark. Finally, fibers were removed, and absorbance was measured at 730 nm using a microplate reader. The percentage of ABTS radical scavenging activity was calculated for both raw and treated fiber samples (Kasote et al., 2019). Similarly, for the PPR assay, A 0.125 mM KMnO_4_ solution was prepared freshly. Then, 1 mL of this solution was added to wells containing control and fiber samples (n = 4) in a microtiter plate and incubated for 5 min. The fibers were then removed, and the absorbance of the plate was measured at 525 nm using a microplate reader. The results were calculated as % PPR activity for both raw and alkali-treated fiber samples (Kasote et al., 2019).

### 2.10 Statistical analysis

All assays were performed using sufficient replicates. Data were expressed as mean ± SD. Microsoft Excel was used for statistical analysis and data visualization.

## 3 Results and discussion

### 3.1 Physicochemical characterization

In this study, raw fibers were extracted from dried *P. gnaphalodes* aerial part by grinding. The yield of fiber obtained was 18.1% ± 2.5%. Later, the effect of alkali treatment (5%) on the physicochemical properties of *P. gnaphalodes* fibers was investigated. The average diameter of raw *P. gnaphalodes* fibers was observed to be 4.68 ± 0.42 μm, which decreased to 4.15 ± 0.40 μm after alkali treatment (Supplementary Figure S1 **)**. This reduction is primarily attributed to the removal of impurities, wax, and extractives from the fiber surface (Pokhriyal et al., 2023). The results chemical composition analysis of raw and alkali-treated *P. gnaphalodes* fibers are documented in Table 1. The composition of natural fibers varies according to their botanical origins, climate, maturity, and extraction method (Chaudhary and Ahmad, 2020). The observed cellulose, hemicellulose, and lignin content of raw *P. gnaphalodes* fibers were similar to that of bamboo, ost, rye, and sugar fibers (Bakar et al., 2020; Karimah et al., 2021). Alkali treatment significantly removed wax, extractives, and hemicellulose from raw fiber. The observed fold loss of hemicellulose, wax, and extractives was 3.9, 4.2, and 9.6 times, respectively. The cellulose content increased by 91.8% after alkali treatment.

**TABLE 1:** The chemical composition of raw and alkali-treated *P. gnaphalodes* fibers.

Constituent	Raw fibers (%)	Alkali-treated fibers (%)
Cellulose	35.5 ± 4.24^a^	68.1 ± 0.91^b^
Hemicellulose	22.8 ± 1.45^a^	5.82 ± 0.88^b^
Lignin	24.5 ± 1.99^a^	20.9 ± 0.00^b^
Pectin	5.82 ± 0.88^a^	3.46 ± 0.29^b^
Wax	4.65 ± 0.13^a^	1.10 ± 0.04^b^
Extractives	6.73 ± 0.71^a^	0.70 ± 0.05^b^
Moisture	8.55 ± 0.29^a^	2.98 ± 0.36^b^
Ash	0.08 ± 0.00^a^	0.06 ± 0.01^b^

The significant differences (*p* ≤ 0.05) among raw and alkali-treated samples are shown by different letters, based on the Student’s t-test.

### 3.2 FTIR analysis

**FIGURE 2:**
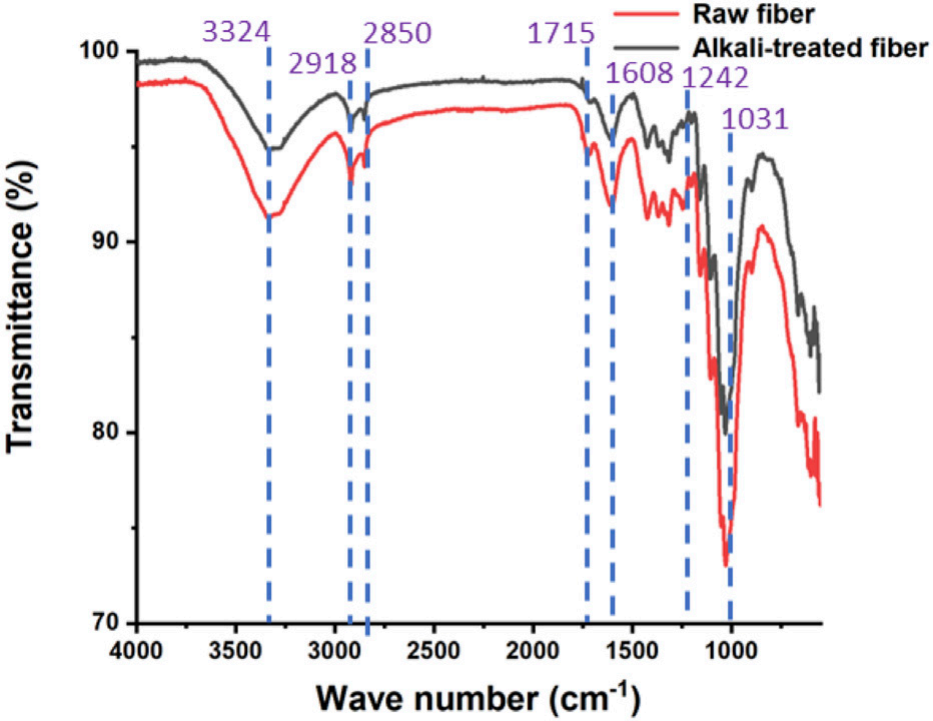
FTIR spectra of the raw and the alkali-treated fibers of *P. gnaphalodes*.

The results of FT-IR analysis of raw and alkali-treated *P. gnaphalodes* fibers are shown in Figure 2. The peak observed around 3,324 cm^−1^ in raw and alkali-treated *P. gnaphalodes* fibers was associated with the stretching vibration of OH groups and inter- and intra-molecular hydrogen bond vibrations. This peak was attributed to the presence of α-cellulose and water molecules due to the moisture content (Merlini et al., 2011; George et al., 2020). Furthermore, the peaks at 2,918 and 2,850 cm^−1^ correspond to the stretching vibrations of C-H bonds in alkanes found in Cellulose I or α-cellulose (Pradhan et al., 2023). The peak at 1715 cm^−1^ corresponds to the C=O stretching vibration in lignin and hemicellulose (Maran et al., 2022). Similarly, the C=O stretching vibration associated with the lignin and hemicellulose components was also confirmed by the peak observed at around 1,608 cm^−1^ (George et al., 2020; Ravindran et al., 2020). The peak at 1,242 cm^−1^ was associated with the C–O stretching vibration of the acetyl group in lignin and hemicellulose (Ponnu Krishnan and Selwin Rajadurai, 2017). However, this peak was almost non-visible in alkali-treated fiber samples, indicating the removal of hemicellulose and lignin from *P. gnaphalodes* fibers during alkali treatment, as reported in previous studies (Kamaruddin et al., 2022). Furthermore, a strong peak at 1,031 cm^−1^ indicated the presence of lignin (C–OH stretching) in the fiber (Senthamaraikannan and Kathiresan, 2018). As seen in the chemical composition analysis, the findings of the FTIR analysis clearly show that a significant amount of hemicellulose, lignin, and other components are removed from the surface of *P. gnaphalodes* fibers during alkali treatment.

### 3.3 XRD analysis

**FIGURE 3:**
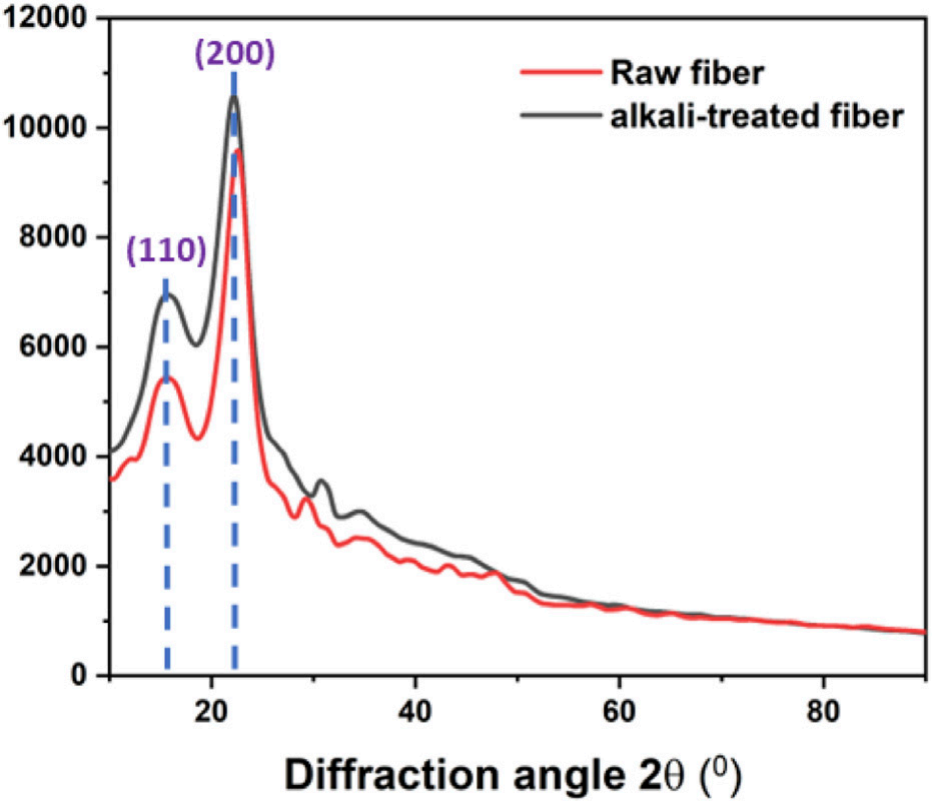
XRD pattern of the raw and the alkali-treated fibers of *P. gnaphalodes*.

The XRD patterns of the raw and alkali-treated fibers of *P. gnaphalodes* are shown in Figure 3. Both raw and alkali-treated fiber samples showed two intense peaks at around 2θ values at 15° and 22°, corresponding to the (110) and (200) planes, respectively. These two peaks at 2θ = 15° and 22° represent the amorphous and crystalline constituents of the fiber, respectively (Rathinavelu and Paramathma, 2023). After the alkali treatment, an increase in the intensities of both peaks was observed, indicating an increase in the crystalline fraction in the alkali-treated fibers. The Crystallinity Index (CI) is a quantitative indicator of crystallinity (Sa et al., 2017).

In general, fibers with a higher CI value may have better mechanical properties (Ravindran et al., 2020). The observed CI of the raw and alkali-treated fibers of *P. gnaphalodes* was 67.0% and 79.2%, respectively, indicating raw fiber had good crystallinity that enhanced considerably with alkali-treatment. This indicates that the raw fiber had good crystallinity, which significantly improved with alkali treatment.

### 3.4 TGA analysis

**FIGURE 4:**
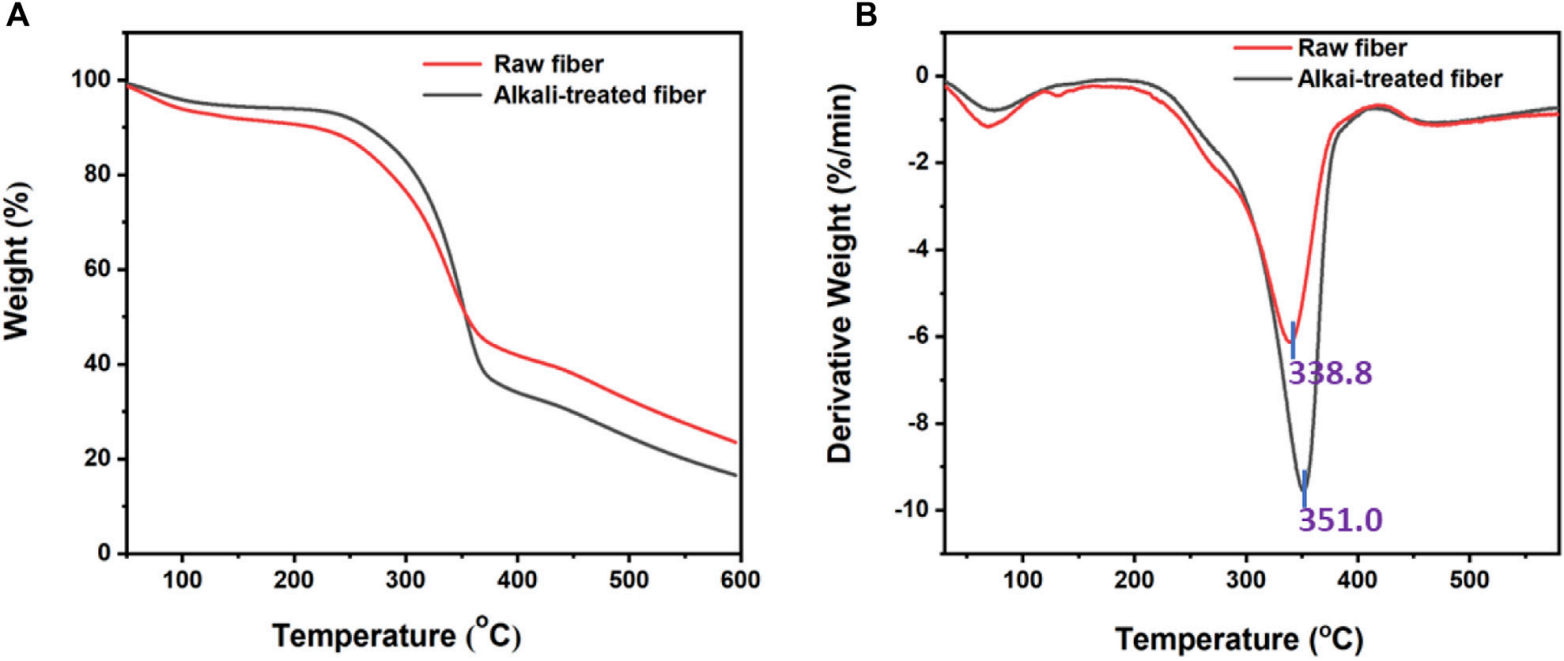
**(A)** TGA and **(B)** DTG graphs of raw and alkali-treated fibers of *P. gnaphalodes*.

The TGA and derivative thermogravimetric (DTG) curves of raw and alkali-treated fibers of *P. gnaphalodes* are shown in Figure 4. In general, studying the thermal degradation properties of natural fibers is crucial for high-temperature applications (Vijay et al., 2019). Both raw and treated samples were found to show weight loss in phases. In the phase (up to 150°C) both raw and alkali-treated fibers showed a small amount of weight loss due to the removal of moisture content in the sample (Borchani et al., 2015). At 150°C, raw and alkali-treated fibers were found to exhibit weight losses of up to 8.14% and 5.58%, respectively, due to their differential moisture content. Next phase, raw (200°C–400°C) and alkali-treated (250°C–400°C) fibers showed maximum weight loss due to the degradation of fiber components such as pectin, hemicellulose, and cellulose (Raia et al., 2021). Typically, lignocellulosic fibers degrade at around 240°C (Mahmud et al., 2021). The observed maximum fiber degradation temperatures for raw and alkali-treated fibers were 338.8°C and 351.0°C, respectively (Figure 4A). This indicates that raw *P. gnaphalodes* fibers have good thermal stability that can be further increased with alkali treatment. In general, alkaline treatment is also used to improve the thermal stability of natural fibers (Elfaleh et al., 2023). Lignins in plant fibers are difficult to degrade, so they begin to break down in the late phase (up to 351.0°C), leaving behind residue (Loganathan et al., 2020). At the end of the experiment, we found that the raw fibers (23.5%) left more residual mass than the alkali-treated fibers (16.5%). This could be due to their high lignin content, including wax.

### 3.5 SEM analysis

**FIGURE 5:**
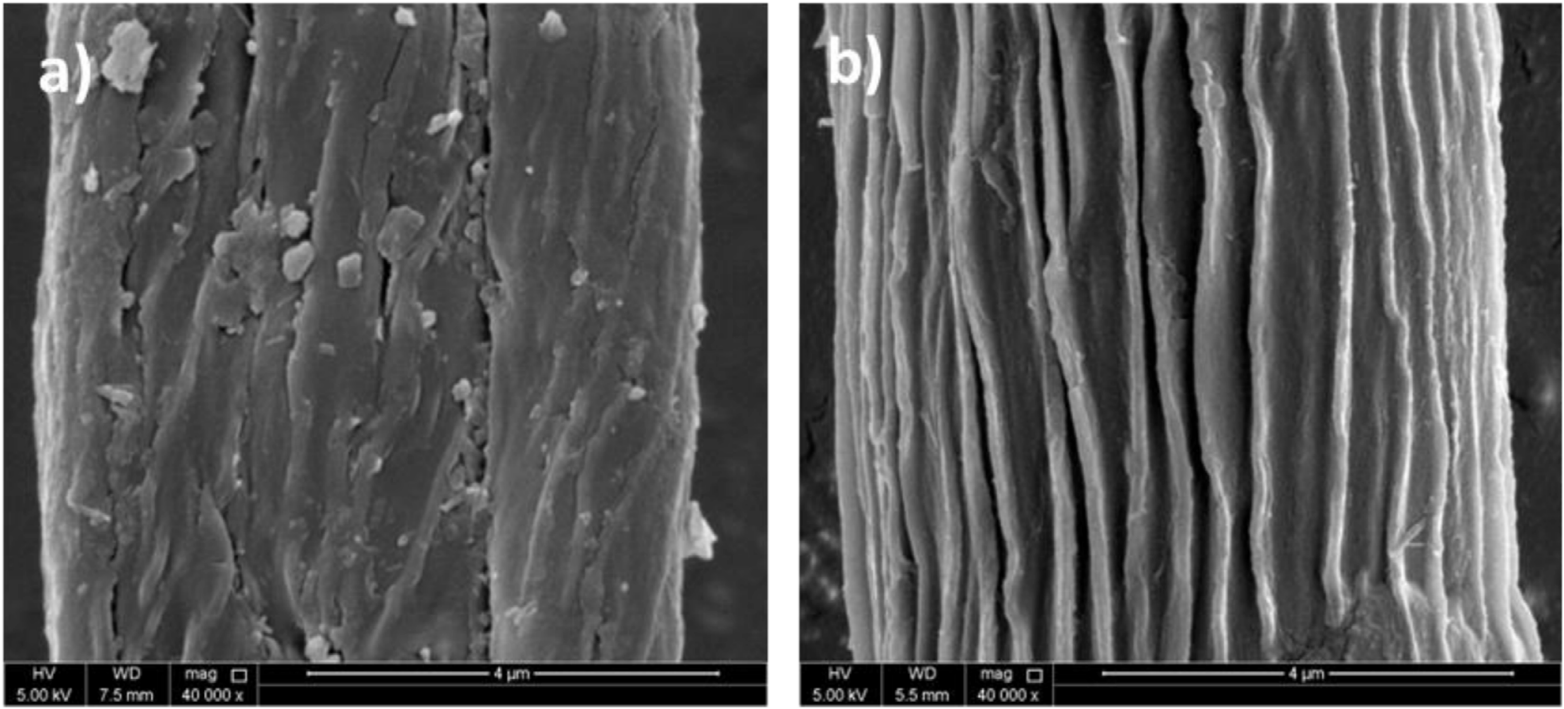
Scanning electron microscopy (SEM) micrographs of **(A)** raw and **(B)** alkali-treated *P. gnaphalodes* fibers.

Figure 5 shows the surface morphology of raw and alkali-treated fibers of *P. gnaphalodes*. The fiber of *P. gnaphalodes* is comprised of a series of parallel microfibrils. However, the surface of the raw fiber was found to be rough and had impurities and wax on it (Figure 5A). After the alkali treatment, impurities, wax, and lignin present on the surface were found to be washed out. Alkali-treated fiber had a smooth surface and clearly visible microfibrils (Figure 5B). This fact was also evidenced by the EDX spectra, which showed a loss of Si, Cl, and Mg contents after alkali treatment (Supplementary Figure S2). These observations collectively indicate that alkali-treated *P. gnaphalodes* fibers can be an appropriate material for making lightweight fiber-reinforced composites.

### 3.6 Antioxidant activity

**FIGURE 6:**
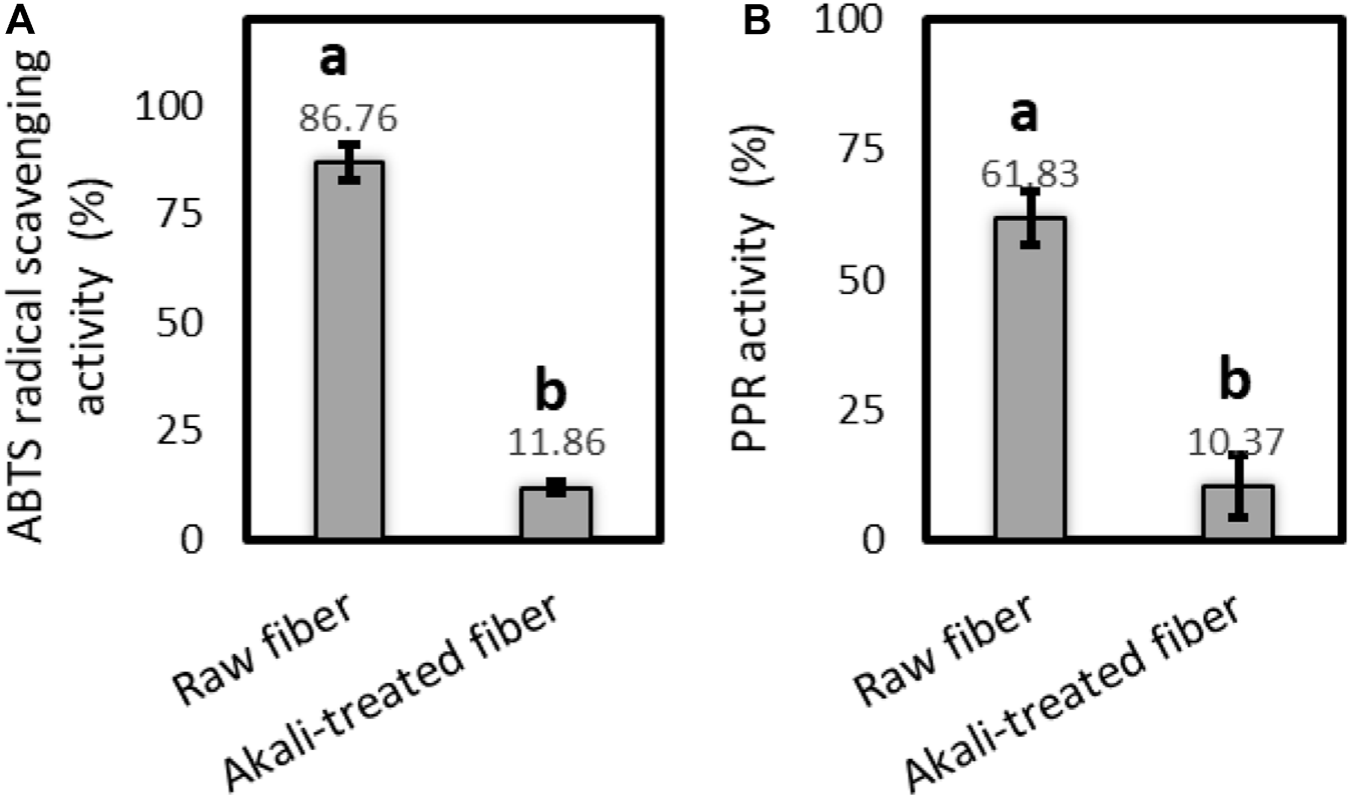
Antioxidant activity of the raw and the alkali-treated fibers of *P. gnaphalodes* in **(A)** ABTS and **(B)** PPR assays.

The results of free radical scavenging and reducing power potentials of the raw and alkali-treated fibers of *P. gnaphalodes* in ABTS and PPR assays are shown in Figure 6. In both assays, the raw fibers of *P. gnaphalodes* showed significantly higher radical scavenging and reducing power potentials compared to the alkali-treated fibers, indicating that the majority of antioxidant components such as lignins and other polyphenols were lost from *P. gnaphalodes* fibers during alkali treatment. We found a nearly 7.3-fold decrease in radical scavenging activity and a 6-fold decrease in reducing power potential in ABTS and PPR assays after alkali treatment, respectively. So far, antioxidant activity has been reported in the flax, hemp and colored cotton fibers (Ma et al., 2016; Zamora-Mendoza et al., 2022; Zamora-Mendoza et al., 2023). These natural fibers with antioxidant properties can function as anti-inflammatory, wound healing, antitumor, and anti-aging agents, as well as prolonging the shelf-life of products (Pedro et al., 2022). These findings about the free radical scavenging and reducing power potentials of raw *P. gnaphalodes* can be utilized in developing functional materials, particularly for cosmetic and wound healing applications.

## 4 Conclusion

In the present study, raw fibers from dried *P. gnaphalodes* aerial parts were extracted by grinding with an average yield of 18.1%. The raw fiber of *P. gnaphalodes* was found to have an average diameter of 4.68 μm, mainly composed of cellulose (35.7%), lignin (24.5%), hemicellulose (22.7%), extractives (6.73%), pectin (5.64%), and wax (4.65%). Furthermore, the effect of 5% alkali treatment on the physicochemical, thermal, morphological, and antioxidant properties was studied. These findings showed that alkali treatment effectively extracted most of the hemicellulose, including lignin, pectin, wax, and extractives, and increased the cellulose content in the fibers. This was further evident from the increased thermal stability of alkali-treated fibers. In antioxidant activity assessment studies, raw fibers of *P. gnaphalodes* showed significantly higher radical scavenging and reducing power potentials compared to the alkali-treated fibers, indicating that the majority of antioxidant components such as lignins and other polyphenols were lost from *P. gnaphalodes* fibers during alkali treatment. Altogether, Overall, this study highlighted that alkali treatment enhanced mechanical and thermal characteristics, including crystallinity. However, the antioxidant activity of raw *P. gnaphalodes* was considerably reduced as a result of this treatment. Moreover, observed promising free radical scavenging and reducing power potentials of raw *P. gnaphalodes* can be utilized in developing functional materials, particularly for cosmetic and wound healing applications.

## Data Availability

The original contributions presented in the study are included in the article/Supplementary Material, further inquiries can be directed to the corresponding author.
