# Supplementary material for: Extraction and characterization of natural fibers from Pulicaria gnaphalodes plant and effect of alkali treatment on their physicochemical and antioxidant properties
Source: Front Chem. 2024 Aug 2;12:1437277. doi: 10.3389/fchem.2024.1437277 (PMC11327012; doi:10.3389/fchem.2024.1437277)
Supplement: Supplementary file 1 [file DataSheet1.docx]

Supplementary Material

**
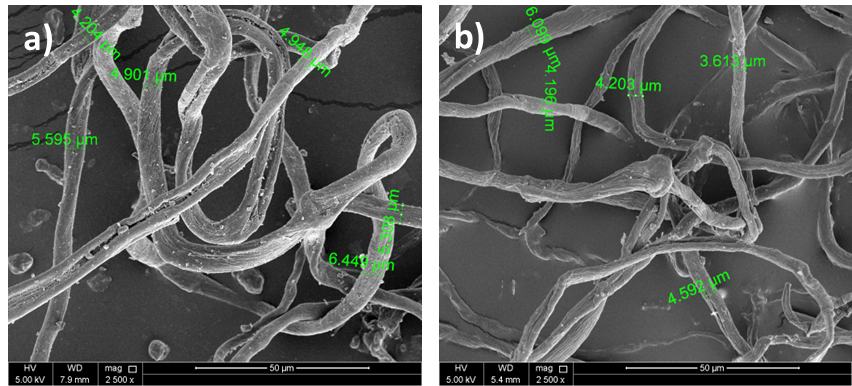
**

**Supplementary Figure 1.** Scanning electron microscopy (SEM) micrographs of (a) raw and (b) alkali-treated *P. gnaphalodes* fibers showing their respective diameters in micrometers (μm).

**
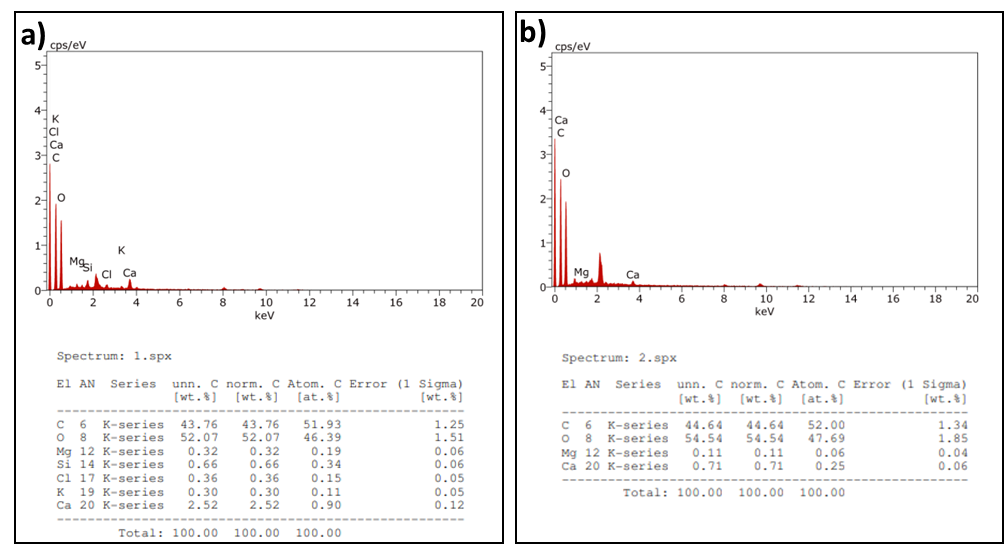
**

**Supplementary Figure 2**. Energy-dispersive X-ray spectroscopy (EDX) spectra of raw and alkali-treated *P. gnaphalodes* fibers
